# *T*-Regulatory Cells and Inflammatory and Inhibitory Cytokines in Malawian Children Residing in an Area of High and an Area of Low Malaria Transmission During Acute Uncomplicated Malaria and in Convalescence

**DOI:** 10.1093/jpids/piu140

**Published:** 2015-01-07

**Authors:** Tonney S. Nyirenda, Malcolm E. Molyneux, Rupert Kenefeck, Lucy S. K. Walker, Calman A. MacLennan, Robert S. Heyderman, Wilson L. Mandala

**Affiliations:** 1 Malawi-Liverpool Wellcome Trust Clinical Research Programme, College of Medicine, Blantyre; 2 Liverpool School of Tropical Medicine, United Kingdom; 3 Institute of Immunity and Transplantation, University College London, Division of Infection and Immunity, United Kingdom; 4 The Medical Research Council Centre for Immune Regulation and Clinical Immunology, Service, School of Immunity and Infection, College of Medicine and Dental Sciences, University of Birmingham, United Kingdom; 5 Basic Medical Sciences Department, College of Medicine, Blantyre, Malawi

**Keywords:** convalescence, IL-10, interleukin-10, lymphopenia, regulatory T cells, uncomplicated malaria

## Abstract

**Background:**

Malaria still infects many Malawian children, and it is a cause of death in some of them. Regulatory T cells (Tregs) help in negating immune-related pathology, it but can also favor multiplication of malaria parasites. The question remains whether children recovering from uncomplicated malaria (UCM) have higher Tregs and interleukin (IL)-10 levels in convalescence.

**Methods:**

We recruited children between the ages of 6 and 60 months presenting with acute UCM in Blantyre (low transmission area) and Chikwawa (high transmission area). We observed the children after 1 month and 3 months and analyzed their blood samples for parasitemia, lymphocyte subsets, and levels of the cytokines interferon (IFN)-γ, IL-10, and transforming growth factor (TGF)-β. Blood samples from age-matched controls were also analyzed for the same parameters.

**Results:**

Compared with controls, acute UCM was associated with mild lymphopenia, splenomegaly, and high levels of IFN-γ, tumor necrosis factor-α, and IL-10, which normalized in convalescence. In Chikwawa, Treg counts were significantly (*P* < .0001) higher in convalescence compared with acute disease, whereas in Blantyre, these were as low as in healthy controls both during acute disease and in convalescence. Blantyre had a higher percentage of parasiteamic children (15% versus 12%) in convalescence compared with Chikwawa, but none of these developed symptomatic malaria during the study duration. Concentrations of TGF-β were higher at time points for the study participants and in controls from Blantyre compared with those recruited in Chikwawa.

**Conclusions:**

The high transmission area was associated with high Tregs counts and IL-10 concentrations in convalescence, which could have an effect on parasite clearance. We recommend that children recovering from UCM, especially those from high transmission area, should sleep under insecticide-treated nets, be screened for parasitemia, and a provision of antimalarial prophylaxis should be considered.

Malaria affects between 300 and 600 million people annually and contributes towards more than 1 million deaths worldwide, with *Plasmodium falciparum* malaria causing more deaths [1–3]. The disease's clinical presentation ranges from asymptomatic parasitemia to febrile disease, presenting as uncomplicated malaria (UCM) or severe and often fatal illness classified either as cerebral malaria (CM) or severe malarial anemia (SMA) [3, 4]. Of the millions of African children who become ill with malaria annually, only 2 percent develop severe disease, and these are the ones who are at greatest risk of dying from the infection [3].

Although factors that determine whether *P falciparum* malaria infection develops into severe disease are not clearly known, current evidence suggests that some interplay between the parasite and host's immune response has a role [5–6]. Successful control and clearance of blood-stage malaria infection requires a well coordinated and timely antibody-mediated and cell-mediated immune response. Cell-mediated immunity involves different cell types that partly exert their effect through the release of proinflammatory cytokines such as interferon (IFN)-γ and tumor necrosis factor (TNF)-α [7, 8].

If the proinflammatory response is left uncontrolled, instead of just achieving the parasite clearance and controlling the parasite replication [9], it can result in the development of immune-mediated pathology [10]. Immunity against severe malaria may depend upon the host's ability to regulate the magnitude and specific timing of the cell-mediated immune response, allowing the sequential induction of appropriate levels of proinflammatory and anti-inflammatory cytokines at crucial stages of the infection cycle [13, 14].

Regulatory T cells (Tregs), a subset of T cells, have been implicated in playing a role in the severity of malaria and also in the clearance of parasites in blood-stage infection in both mouse models and in human malaria [14, 15]. Depletion of Tregs protected mice from death when infected with a lethal strain of *Plasmodium yoelii* [16]. Both in vitro and in vivo depletion of Tregs significantly reversed the inhibition of interleukin (IL)-2 production, a proinflammatory cytokine, in *Plasmodium berghei*-infected mice [17].

Studies in humans have shown that Treg levels increase during acute *P falciparum* malaria infection coinciding with increased parasite growth rate [21], which is thought to be essential in controlling proinflammatory responses in subsequent malaria infections, thereby preventing development of severe malaria [22].

A longitudinal study conducted in Kenya found an association between the percentage of Tregs and risk of subsequent clinical malaria upon reinfection [24]. A separate longitudinal study of healthy Gambian children and adults in high malaria transmission areas found that percentage and absolute numbers of Tregs increased significantly during the malaria season but were low in the dry season [27]. Taken together, the findings of these studies seem to suggest that pre-existing levels of Tregs may influence malaria susceptibility and severity. In a recent study, Gambian children presenting with acute UCM and SM were reported to have higher percentages and numbers of Tregs during convalescence compared with controls [14]. In addition, children living in endemic malaria areas would usually have suffered from UCM several times in the previous years before succumbing to CM [20].

We therefore conducted the current study to compare and contrast Tregs and cytokine levels in children presenting with UCM at a location with a high (Chikwawa) and a location with a low (Blantyre) malaria transmission rate.

## METHODS

### Study Site and Participants

We conducted a prospective cohort study at Ndirande Health Centre (NHC) in Blantyre (area of low malaria transmission [32]) between March 2011 and July 2011 and at Chikwawa District Hospital (CDH) in Chikwawa (area of high malaria transmission [32]) between August 2011 and November 2011. We recruited 33 and 30 human immunodeficiency virus (HIV)-uninfected children aged between 6 and 60 months in Blantyre and Chikwawa, respectively, who presented with acute UCM. Participants were defined as having UCM when they were febrile at the time of recruitment, had a positive Rapid Diagnostic Test or positive malaria thick and thin slides, but had a Blantyre Coma Score of 5, and a hemoglobin (Hb) concentration above 5 dL/mL [4]. Children who were HIV-infected or presented with other comorbidities, infectious or noninfectious, were excluded from the study. Children with UCM were observed at 1 month and 3 months during convalescence. In addition, 30 healthy controls of ages that were in the same range as the study participants (ie, between 1 and 60 months) were recruited at both sites. Healthy controls were recruited from NHC and CDH under the following conditions: when they came for scheduled immunization and were not febrile; they had a temperature of 37°C or less when enrolled; they had not had clinical- or laboratory-confirmed malaria in the past 3 months; and they were HIV-uninfected and did not have any underlying illness at the time of recruitment. Thick and thin malaria slides were prepared from the blood sample of each healthy control on the day of recruitment to screen for malaria parasitemia.

A 5 mL venous blood sample was collected from each participant at recruitment and during follow-up. Participants presenting with UCM were treated with coformulated tablets of 20 mg of artemether and 120 mg of lumefantrine (Coartem; Novartis), per Malawi Government guidelines as first-line treatment of UCM, before blood sample collection. Ethical approval for the study was obtained from College of Medicine Research and Ethics Committee, and written informed consent was obtained from the parent or guardian of every participant.

### Human Immunodeficiency Virus, Full Blood Count, and Malaria Parasite Testing

Human immunodeficiency virus testing was performed using 2 rapid test kits; Determine (Abbott Laboratories, Japan) and Unigold (Trinity Biotch, Dublin). Where discordant results were obtained, polymerase chain reaction was used to confirm the results. Thick and thin blood smears on slides were prepared by standard methodology. Total white blood cell (WBC) counts, percentage, and absolute counts of lymphocytes were determined using an HmX hematological analyzer (Coulter).

### Immunophenotyping Procedure

**Figure 1.**
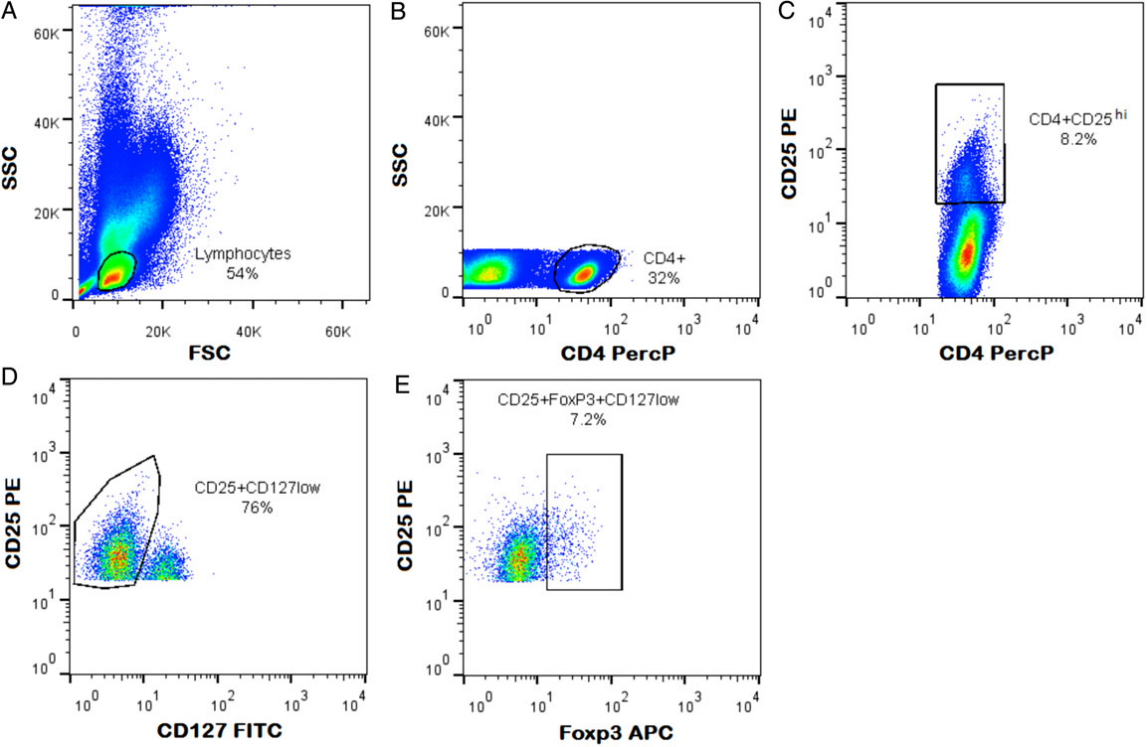
Gating strategy for CD4^+^CD25^hi^FoxP3^+^CD127^low^ regulatory T cells that was used in this study. FITC, fluorescein isothiocyanate; PercP, peridinin chlorophyll protein.

Immunophenotyping of blood samples by flow cytometry and lymphocyte subset identification was performed as reported elsewhere [31]. A total of 200 µL of whole blood was stained with a cocktail of conjugated surface monoclonal antibodies (mAbs): CD4 peridinin chlorophyll 4 µL, CD25 phycoerythrin 4 µL, and CD127 fluorescein isothiocyanate 4 µL (all from Beckon Dickinson) were incubated for 15 minutes at room temperature in the dark. Erythrocytes were lysed with 2 mL 1:10 diluted FACS lysing solution (Becton Dickinson), incubated in the dark for 20 minutes. Cells were washed twice with 2 mL phosphate-buffered saline ([PBS] Sigma-Aldrich) and fixed with 500 µL 1:3 diluted Perm/Fix solution (Becton Dickinson), incubated for 20 minutes at room temperature in the dark. Cells were washed twice with 2 mL 1:10 diluted Perm Buffer (Beckon Dickinson). Permeabilized cells were stained with intracellular conjugated monoclonal antibody: 4 µL FoxP3 APC (Beckon Dickinson) for 30 minutes at room temperature in the dark and washed with 2 mL of Perm Buffer. Stained cells were resuspended in 350 µL 1% formaldehyde (Sigma-Aldrich)/PBS and analyzed on a CyAn flow cytometer (Beckman Coulter) within 24 hours. Figure 1 shows the gating strategy used for classic Tregs [33]. We defined CD4^+^ Tregs as a subset that expressed CD4^+^CD25^hi^FoxP3^+^CD127^low^.

### Quantification of Serum Cytokine Levels

High protein binding 96-well enzyme-linked immunosorbent assay (ELISA) plates (Nunc-Immuno) were coated with monoclonal antibodies for IFN-γ, TNF-α, IL-10, and transforming growth factor (TGF)-β: 1-D1 K, TNF3/4, 9D7, and MT593 (all from MABTECH AB), respectively, at 2 µg/mL in PBS (Sigma-Aldrich) and adding 100 µL per well. Coated ELISA plates were incubated overnight at 4°C and washed twice with PBS adding 200 µL per well. Plates were blocked with PBS with 0.05% Tween 20 containing 0.1% bovine serum albumin (all from Sigma-Aldrich) (incubation buffer) and incubated at room temperature for 1 hour. Enzyme-linked immunosorbent assay plates were washed 5 times with PBS containing 0.05% Tween 20. A total of 100 µL of test serum and standards were added to appropriate wells, diluted in incubation buffer, and incubated at room temperature for 2 hours. Enzyme-linked immunosorbent assay plates were washed 5 times with PBS containing 0.05% Tween 20 and appropriate biotinylated mAbs for detection of serum IFN-γ, TNF-α, IL-10, and TGF-β: 7-B6-1-biotin, mAb TNF5-biotin, mAb 12G8-biotin, mAb MT517-biotin (MABTECH AB), respectively, at 1 µg/mL were added at 100 µL per well and incubated at room temperature for 1 hour. Enzyme-linked immunosorbent assay plates were washed 5 times with PBS containing 0.05% Tween 20 and 100 µL Streptavidin-ALP diluted 1:1000 (MABTECH AB) in incubation buffer added per well and incubated at room temperature for 1 hour. Enzyme-linked immunosorbent assay plates were washed 5 times with PBS containing 0.05% Tween 20 and 100 µL SIGMAFAST *p*-Nitrophenyl phosphate (Sigma-Aldrich) per well, and optical density was measured after 30 minutes of incubation using BioTek reader ELx800 (BioTek Instruments) at 405 nm. Concentrations of serum cytokines were determined using plotted standard curve.

### Spleen Size Grading

Spleen size grading was performed using a protocol described previously [28]. In brief, the size of the spleen was examined while the child was resting supine with both hands at the side. The examiner left hand was used to support the left of the ribcage posterior laterally while the right hand was aligned with the fingertips parallel to the left costal margin. Palpation was done from the right lower quadrant towards the left costal margin, asking the patient to take a deep breath in and feeling for the movement of the spleen with the examiners’ fingers. A spleen that was felt was graded using the Hackett's grading system [28]. Essentially, the higher the spleen size grade the larger the spleen.

### Statistical Analyses

Statistical analysis was performed using GraphPad Prism. A Mann-Whitney *U* test was performed between groups to detect statistically significant differences. A *P* value of < .05 was considered to show a statistically significant difference. Regression analysis was used to assess linear associations between the concentration of IL-10 and the percentage of Tregs at the 2 sites, with the Spearman's correlation value (r) of 1 indicating a strong correlation and anything lower showing a weaker correlation.

## RESULTS

### Participants' Demographic Data

In Blantyre, 33 children presenting with UCM were recruited with a median age of 34.5 months (5.7–60). Twenty-seven of these children were observed at 1 month convalescence, and 26 children were successfully observed again after 3 months (Table 1). Of those not seen during the 2 follow-up visits, 5 withdrew from the study mainly because their parents or guardians did not want their children to continue participating in the study, and 1 died of causes other than malaria. In Chikwawa, 30 children presenting with acute UCM and with a median age of 26.9 months (6.1–51.3) were recruited; 5 withdrew from the study 1 month after recruitment, and 1 additional child withdrew by the second follow-up visit for the same reason as in Blantyre. Although all UCM participants recruited in Blantyre had positive parasitaemia on the day of recruitment, 4 participants were found to be parasitemic during convalescence by thick and thin malaria slides, whereas in Chikwawa, 3 participants were parasitemic after 3 months convalescence.

**Table 1.** Demographic, Hematological, and Spleen Grade Data for the Study Participants in Blantyre and Chikwawa

Study Participants' Parameters	Blantyre				Chikwawa			
	Acute	1 Month	3 Months	Control	Acute	1 Month	3 Months	Control
Number of participants (%)	33	27	26	31	30	25	24	30
Median age in months (range)	34.5 (5.7–60)	ND	ND	16.5 (6.3–55.2)	26.9 (6.1–51.3)	ND	ND	24.8 (9.9–46.8)
Loss to follow up (%)	NA	2 of 33 (6)	0 of 27 (0)	NA	NA	5 of 30 (16.6)	1 of 25 (4)	NA
Number of deaths (%)	NA	1 of 28 (3.5)	0 of 26 (0)	NA	NA	0 of 25 (0)	0 of 24 (0)	NA
Malaria positive slide or RDT (%)	33 of 33 (100)	4 of 27 (14.8)	4 of 26 (15.4)	0 of 31 (0)	30 of 30 (100)	3 of 25 (12)	3 of 24 (12.5)	0 of 30 (0)
Median Hgb in g/dL (range)	9.2 (4.9–12.1)	11 (5.9–13.4)	11.6 (6.6–13.7)	10.1 (5.6–14)	8.9 (6.4–11.9)	10.7 (3.1–12.8)	11 (8.2–12.8)	10.7 (3.1–12.8)
Median RBC ×10^6^/μL (range)	3.9 (2.3–5)	4.2 (3.5–5.8)	4.4 (2.5–8.7)	4.5 (2.9–7.3)	3.5 (2.1–3.9)	4.1 (2.8–5)	4.4 (3.6–5.5)	4.5 (1.0–5.7)
Median WBC ×10^3^/μL (range)	9.4 (4.6–22.4)	9.4 (3.7–23)	7.2 (5.5–10.3)	8.5 (3.9–17.4)	7.7 (3.9–18.5)	9.6 (5.0–14.7)	8.2 (5.3–15.2)	9.1 (4–14.8)
Number of spleen grade 0 (%)	ND	ND	21 of 26 (80.7)	27 of 31 (90)	10 of 30 (33.3)	22 of 25 (88)	24 of 24 (100)	28 of 30 (93.3)
Number of spleen grade 1 (%)	ND	ND	2 of 26 (7.6)	3 of 31 (10)	16 of 30 (53.3)	2 of 25 (8)	0 of 24 (0)	2 of 30 (6.6)
Number of spleen grade 2 (%)	ND	ND	3 of 26 (11.5)	0 of 31 (0)	4 of 30 (13.3)	1 of 25 (4)	0 of 24 (0)	0 of 30 (0)

Abbreviations: Hgb, hemoglobin; NA, not applicable; ND, not done; RBC, red blood cells; RDT, rapid diagnostic test; WBC, white blood cells.

### Acute Uncomplicated Malaria Was Characterized by Anemia and Splenomegaly

Acute malaria was characterized by significantly lower median Hb levels as well as red blood cell counts compared with controls at both sites (*P* < .005). Both of these parameters normalized during convalescence (Table 1). A complete data set for spleen grades was only collected from the participants in Chikwawa because this assessment was initiated after recruitment had already been completed in Blantyre. The majority of the acute malaria cases had a spleen grade 1 (53%), followed by grade 0 (33%) with as high as 13% qualifying for grade 2. This is consistent with the hypothesis that acute malaria is associated with splenomegaly. By the second follow up, the spleen grades of all malaria cases had normalized, suggesting that the splenomegaly observed during acute malaria is transient. In addition, as expected, at both sites none of the controls had a spleen grade of 2, and the majority of the controls had a spleen grade of 0 (Table 1).

### Absolute Tregs Counts Were Higher During Convalescence at the High Transmission Site

**Figure 2.**
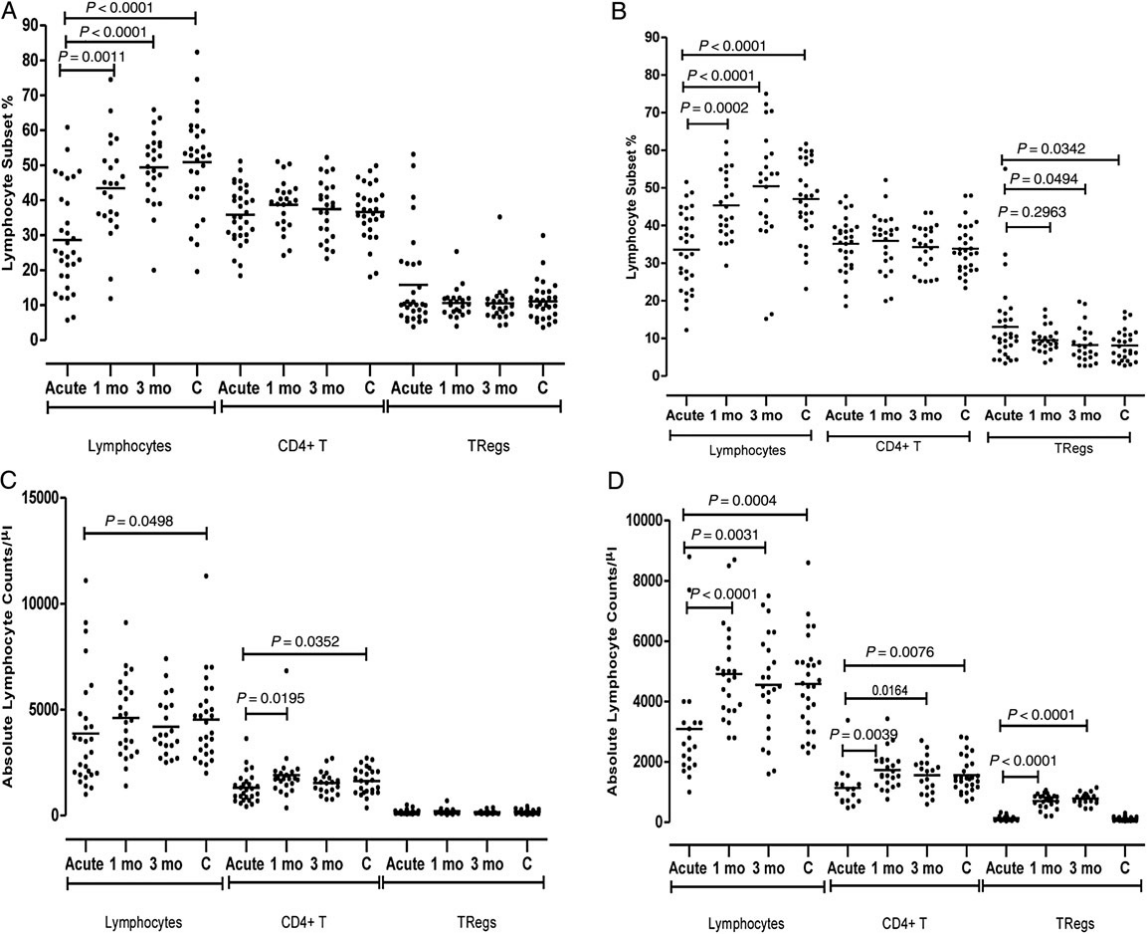
Proportions of median values of lymphocytes, CD4^+^ T cells and regulatory T cells (Tregs) at different stages of infection in children recruited with uncomplicated malaria and in healthy controls presented as percentage for the Blantyre (A) and Chikwawa (B) sites and presented as absolute counts for the Blantyre (C) and Chikwawa (D) sites.

When lymphocytes were presented as a percentage of total WBCs (Figure 2A and B) and when they were presented as absolute counts (Figure 2C and D), acute UCM was characterized by lymphopenia, which normalized in convalescence. CD4^+^ T cell-specific lymphopenia in acute UCM was only significant when the subset was presented as absolute counts at both sites, and this too normalized during convalescence (Figure 2C and D). When presented as percentages of total CD4^+^ T cells, acute UCM was characterized by higher than normal levels of Tregs, although the difference between levels observed during acute malaria and those in controls was only significant in Chikwawa (*P* = .0342) (Figure 2A and 2B). Regulatory T cell percentages significantly decreased in Chikwawa from the high levels observed during acute infection to low levels during the second follow up (*P* = .0494). Although a similar trend was observed in Blantyre, the decrease in percentage Tregs during convalescence was not significant.

However, when the number of cells expressing the Tregs phenotype was calculated using lymphocyte counts from the differential WBC, there were no differences in the Tregs counts between acute stage and any of the convalescence stages and the controls in Blantyre (Figure 2C), whereas in Chikwawa, Tregs counts were significantly higher (*P* < .0001 at both 1-month and 3-month stages) in convalescence compared with the cell counts in acute infection (Figure 2D).

Compared between sites, healthy controls in Blantyre had significantly higher percentages (*P* = .0247) and absolute counts (*P* = .0210) of Tregs compared with the controls in Chikwawa (Table 2).

**Table 2.** Comparison of the Medians (Range) of Various Parameters Between Participants Recruited in Blantyre and Those Recruited in Chikwawa

Group	%Lymphocytes	Lymphocyte Count/μL	% CD4^+^ T Cell	CD4^+^ T-Cell Counts/μL	%Tregs	Tregs Cell Counts/μL	IFN-γ (pg/mL)	TNF-α (pg/mL)	IL-10 (pg/mL)	TGF-β (pg/mL)
Blantyre (acute)	25.05 (5.71–60.90)	3240 (1000–11,090)	35.27 (18.41–51.15)	1213 (424–3636)	10.26 (3.79–53.13)	138 (71–499)	174 (64–1776)	571 (146–2948)	355 (56–1500)	205 (56–731)
Chikwawa (acute)	34.05 (12.20–65.99)	2550 (1000–8800)	36.15(18.62–47.78)	1020 (478–3379)	10.32 (3.38–55.05)	107 (39–328)	162 (69–870)	574 (204–4286)	313 (70–947)	80 (14–173)
*P* value	0.0928	0.3205	0.6916	0.3461	0.5985	0.1163	0.5433	0.7336	0.7432	**< 0.0001**
Blantyre (1 month)	44.73 (11.85–74.51)	4500 (1400–9100)	39.48 (24.23–51.07)	1733 (358–6829)	10.21 (3.99–25.37)	169 (43–697)	159 (69–985)	1431 (310–4508)	113 (68–787)	147 (41–396)
Chikwawa (1 month)	43.71 (29.34–62.26)	4850 (2800–8700)	37.61 (19.94–52.10)	1566 (760–3430)	9.15 (3.56–17.69)	788 (205–1065)	100 (61–262)	656(166–4134)	77 (41–279)	101 (20–248)
*P* value	0.4892	0.5417	0.1479	0.6911	0.3121	**< 0.0001**	0.0957	0.1447	**0.0017**	**0.0014**
Blantyre (3 months)	50.67 (19.98–65.90)	3800 (2500–7400)	38.45 (23.31–52.24)	1550 (769–2706)	9.605 (4.18–35.24)	134 (61–369)	174 (58–1318)	728 (212–3012)	87 (49–773)	180(81–731)
Chikwawa (3 months)	52.40 (15.18–75.02)	4500 (1600–7500)	35.20 (25.12–43.45)	1689 (598–2713)	6.68 (2.75–19.77)	813 (452–1150)	101 (59–311)	679 (350–3478)	87 (46–279)	93(20–149)
*P* value	0.7739	0.4062	0.1281	0.8331	0.0597	**0 < 0.0001**	0.2041	0.5288	0.6817	**< 0.0001**
Blantyre (controls)	52.81 (19.58–82.81)	4400 (2000–11,300)	37.02 (18.05–49.86)	1,640 (361–2,709)	9.99 (3.63–29.92)	166 (41–439)	217 (79–1,142)	783 (188–2,762)	88 (59–329)	232 (99–1088)
Chikwawa (controls)	47.78 (23.17–61.73)	4550 (2300–8600)	32.78 (23.37–47.94)	1447 (701–2828)	6.72 (2.76–17.06)	134 (39–305)	105 (59–723)	654(254–2,992)	107 (46–256)	133 (9–760)
*P* value	0.2135	0.6553	0.0548	0.6625	**0.0247**	**0.0210**	**0.0448**	0.8286	0.7681	**0.0005**

Abbreviations: IFN, interferon; IL, interleukin; TGF, transforming growth factor; TNF, tumor necrosis factor; Tregs, regulatory T cells.

The *P* values presented in bold are the ones that showed significant differences when specific parameters were compared between the two sites.

### Acute Uncomplicated Malaria Was Characterized by High Concentrations of Pro- and Anti-inflammatory Cytokines

**Figure 3.**
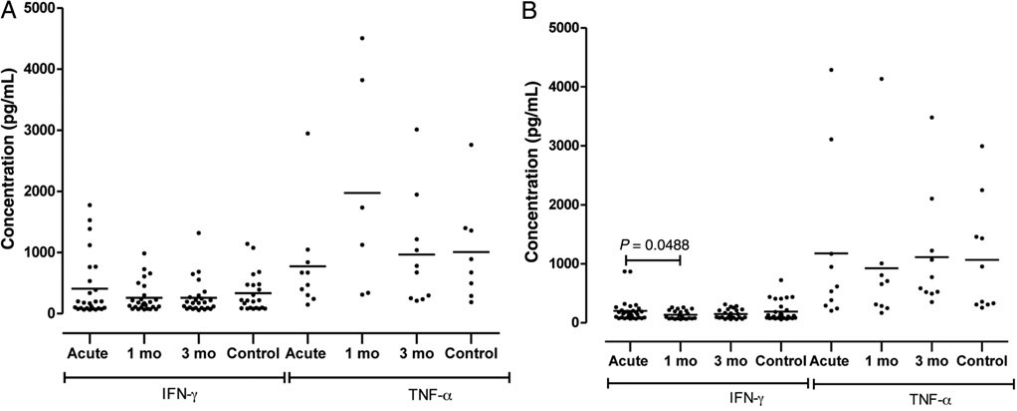
Concentrations of interferon (IFN)-γ and tumor necrosis factor (TNF)-α in serum of sera collected at different stages of uncomplicated malaria infection and in healthy controls in Blantyre (A) and Chikwawa (B).

**Figure 4.**
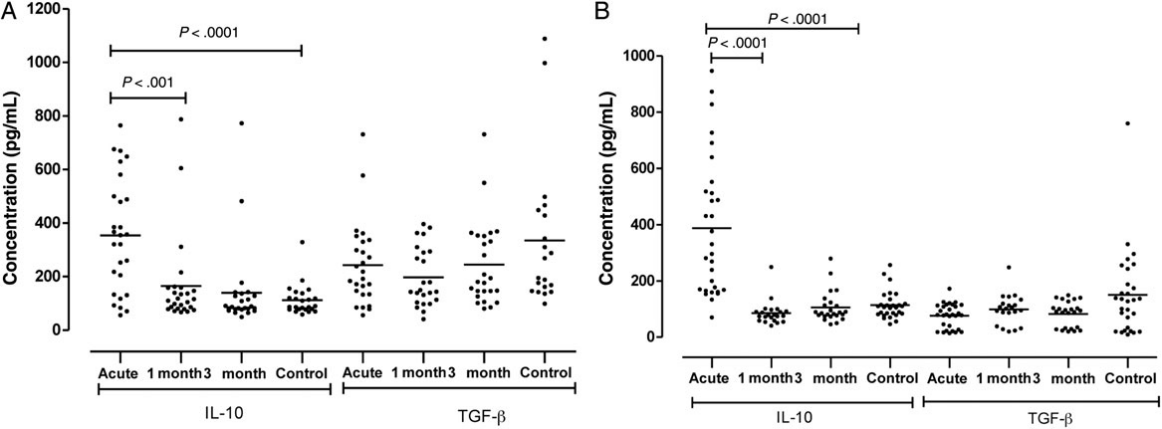
Concentrations of interleukin (IL)-10 and transforming growth factor (TGF)-β in sera collected from children presenting with uncomplicated malaria at different stages of infection and in controls in Blantyre (A) and in Chikwawa (B).

Participants presenting with acute malaria at both sites were characterized by high levels of IFN-γ compared with the controls (*P* = .2683 for Blantyre and *P* = .7485 for Chikwawa) (Figure 3). The higher IFN-γ levels then normalized by the first month in convalescence at both sites, but the decrease was only significant among Chikwawa participants (*P* = .2641 for Blantyre and *P* = .0488 for Chikwawa). Levels of TNF-α (Figure 3) and TGF-β (Figure 4) did not differ between the 4 groups.

Serum from children presenting with acute malaria at both sites had significantly higher levels of IL-10 compared with that of controls (*P* < .0001 for both sites) (Figure 4), and these high levels decreased significantly to normal levels during follow-up period even as early as 1 month after recruitment (*P* = .0008 for Blantyre and *P* < .0001 for Chikwawa).

When compared between sites, at 1 month convalescence stage, study participants in Blantyre had significantly higher concentrations of IL-10 (*P* = .0017) and TGF-β (*P* = .0014) compared with participants in Chikwawa (Table 2). Study participants in Blantyre had significantly higher levels of TGF-β compared with study participants in Chikwawa at all stages (*P* < .0001 in acute and at 3-month stage, *P* = .0014 at 1-month stage). Healthy controls in Blantyre had significantly higher concentrations of IFN-γ (*P* = .0448) and TGF-β (*P* = .0005) compared with healthy controls in Chikwawa (Table 2).

### Percentage of Tregs Correlates Strongly With Interleukin-10 Levels in Acute Disease

**Figure 5.**
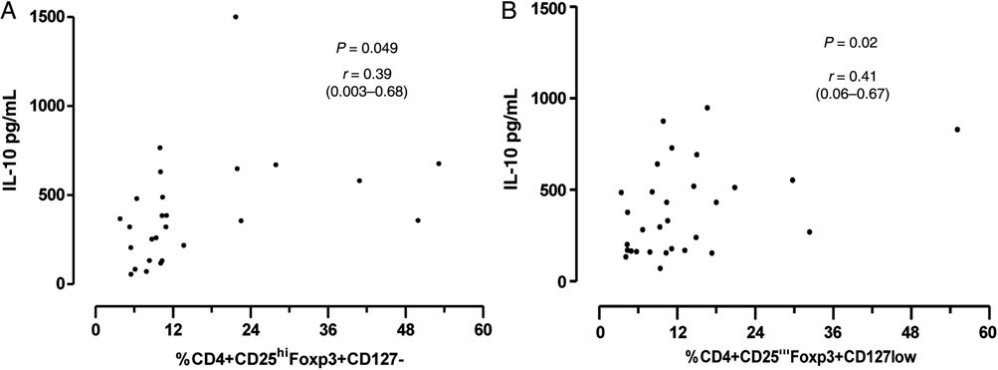
Correlation of regulatory T cells and interleukin (IL)-10 in samples collected from Blantyre (A) and Chikwawa (B). r is the Spearman's correlation, and the 95% coefficient interval is provided in the brackets.

There was significant linear correlation between the IL-10 levels and the percentage of Tregs (Figure 5) during the acute infection at both sites (Chikwawa r = 0.40, *P* = .02, Blantyre r = 0.389, *P* = .049), suggesting that Tregs are an important producer of IL-10.

## DISCUSSION

We found that children recruited with acute UCM presented with lymphopenia and splenomegaly and higher than normal levels of IFN-γ, TNF-α, and IL-10, which normalized 1 month into convalescence. We also found that 15% of the children recruited with acute UCM were parasitemic during convalescence, but none of these participants developed any further form of symptomatic malaria. When Tregs were presented as percentages of the CD4^+^ T cells, we found that they were significantly higher in acute UCM compared with levels in healthy controls only at Chikwawa site. These then normalized in convalescence with the difference being significant only during the second follow up. Although a similar trend was observed in Blantyre, the differences were not significant between any stages.

However, when the levels of Tregs were presented as absolute counts, we found that these were just as low in acute malaria as in healthy controls at both sites (Figure 2C and D), consistent with the results of a study conducted in Peru Amazon that reported low levels of Tregs in asymptomatic individuals and in acute symptomatic malaria [30]. In Blantyre, the low transmission area, the Tregs counts did not differ between any of the groups and stages (Figure 2C); however, in Chikwawa, the high transmission area, the Tregs counts were significantly (*P* < .0001) higher in convalescence compared with acute disease (Figure 2D). This observation is consistent with the results of a study conducted in The Gambia [14], but it is different from our study. The Gambian study found no differences between acute and convalescent values when the Tregs levels were presented as percentages. In the case of the Tregs percentage results of our study, when the results of a few outliers were not included in the analysis, the percentages for acute UCM were similar to those observed in convalescence and in controls. However, revisiting the clinical and demographic characteristics of those few outliers did not isolate anything special about them compared with the other study participants.

Healthy controls in Blantyre were found to have significantly higher percentages and counts of Tregs compared with those from Chikwawa. Acute UCM participants in Blantyre had almost identical levels, but these ended up having significantly lower levels during convalescence (Table 2). The investigators of the 2 Gambian studies [14, 26] had argued that acute malaria triggers expansion of Tregs, which end up persisting for some weeks to maintain immune homeostasis during the contraction phase of the effector response. This could explain the higher than normal percentages and counts of Tregs in participants from the high transmission area of Chikwawa during convalescence.

The high levels of the proinflammatory cytokines IFN-γ and TNF-α during acute UCM have been observed in other studies before [4, 28]. This is consistent with the current understanding that high levels of these proinflammatory cytokines are required for the clearance of parasitemia at the early stage of the infection [5]. Ideally, this predominantly proinflammatory environment is supposed to be followed by the release of high levels of anti-inflammatory cytokines such as IL-10 and TGF-β, although this could favor further parasite maturation and multiplication. Therefore, the observed high levels of IL-10 during acute infection in our study have been described as ideal and are consistent with results of other studies [13, 28]. However, our study was not able to replicate results of studies that have shown significantly higher than normal levels of TGF-β in acute UCM [21].

We had hypothesised that children presenting with acute UCM would have higher levels of the anti-inflammatory cytokines IL-10 and TGF-β, which would remain elevated compared with levels in healthy controls into convalescence. We had therefore anticipated that this predominantly anti-inflammatory profile would render the children more vulnerable to re-infection with malaria and to an increased probability of such re-infection developing into a more severe clinical form of malaria. In contrast, we found that at both sites, the levels of the cytokines IL-10 and TGF-β were much lower in convalescence compared with those observed during acute disease, and they were comparable to those levels observed in healthy controls. Although some participants at both sites were found to be asymptomatically parasitemic 1 month after treatment (14.8% in Blantyre and 12% in Chikwawa), and even more at the 3-month follow-up stage, none of these participants developed any symptomatic malaria during the 3-month period of convalescence.

The observed lower IL-10 levels in healthy controls compared with acute disease is consistent with results found in Malian children [28], adding weight to the hypothesis that IL-10 is required to negate immune-mediated pathology associated with high levels of proinflammatory cytokines such as IFN-γ, TNF-α, and IL-2 [5, 9]. The positive correlation between Tregs and IL-10 levels we have shown in this study is also consistent with what other studies have shown, both in mice models [30] and in humans [14], suggesting that Tregs are one of the main producers of the anti-inflammatory cytokine IL-10. Although we did not find a similar correlation between Tregs and levels of TGF-β in acute UCM, others have shown that levels of this cytokine also increase significantly during acute disease [21].

We did not expect to find any child having parasitemia during convalescence at both sites because we anticipated that the antimalarial treatment would clear all parasites from the infected children. Although a majority of the parents and guardians of the study participants reported to be using insecticide-treated nets during convalescence period, we did not determine whether this parasitemia resulted from new infection or was due to the children failing to completely clear the initial parasitemia. These results need to be replicated in another study, nevertheless such parasitaemia prevalence post treatment might justify administering an additional antimalarial prophylaxis in the affected individuals.

## CONCLUSIONS

Further studies need to be done to establish the role of Tregs in the transition from UCM to the more severe clinical forms of malaria, namely CM and SMA. In addition, by having larger sample sizes and observing the study participants for as long as 1 year or even 2 years, researchers can provide a better understanding of Tregs perturbations at times when the children are purely parasitemic and asymptomatic, when they develop UCM, and when they transition from UCM to the life-threatening CM. In addition, with malnutrition known to affect cytokine production and T-cell differentiation [34], subsequent studies will have to take nutrition status of the participants into consideration. Although anorexia nervosa, an extreme form of dietary calorie restriction, was shown not to affect Tregs and cytokine profile of the affected participants [35], knowing the nutrition status of study participants could also rule out nutrition as a confounder in future studies.
